# Normal Fertility Requires the Expression of Carbonic Anhydrases II and IV in Sperm*

**DOI:** 10.1074/jbc.M115.698597

**Published:** 2015-10-20

**Authors:** Petra M Wandernoth, Nadja Mannowetz, Jaroslaw Szczyrba, Laura Grannemann, Anne Wolf, Holger M. Becker, William S. Sly, Gunther Wennemuth

**Affiliations:** From the ‡ Institute of Anatomy, University Hospital, University Duisburg-Essen, Hufelandstraße 55, 45122 Essen, Germany,; the § Department of Internal Medicine I, Saarland University Medical Center, Kirrberger Straße, 66421 Homburg/Saar, Germany,; the ¶ Division of Zoology/Membrane Transport, Department of Biology, University of Kaiserslautern, Erwin-Schrödinger-Straße 13, 67663 Kaiserslautern, Germany, and; the ‖ Edward A. Doisy Department of Biochemistry and Molecular Biology, St. Louis University School of Medicine, St. Louis, Missouri 63104

**Keywords:** bicarbonate, carbon dioxide, fertilization, mutant, sperm, carbonic anhydrase, subfertility

## Abstract

HCO_3_^−^ is a key factor in the regulation of sperm motility. High concentrations of HCO_3_^−^ in the female genital tract induce an increase in sperm beat frequency, which speeds progress of the sperm through the female reproductive tract. Carbonic anhydrases (CA), which catalyze the reversible hydration of CO_2_ to HCO_3_^−^, represent potential candidates in the regulation of the HCO_3_^−^ homeostasis in sperm and the composition of the male and female genital tract fluids. We show that two CA isoforms, CAII and CAIV, are distributed along the epididymal epithelium and appear with the onset of puberty. Expression analyses reveal an up-regulation of CAII and CAIV in the different epididymal sections of the knockout lines. In sperm, we find that CAII is located in the principal piece, whereas CAIV is present in the plasma membrane of the entire sperm tail. CAII and CAIV single knockout animals display an imbalanced HCO_3_^−^ homeostasis, resulting in substantially reduced sperm motility, swimming speed, and HCO_3_^−^-enhanced beat frequency. The CA activity remaining in the sperm of CAII- and CAIV-null mutants is 35% and 68% of that found in WT mice. Sperm of the double knockout mutant mice show responses to stimulus by HCO_3_^−^ or CO_2_ that were delayed in onset and reduced in magnitude. In comparison with sperm from CAII and CAIV double knockout animals, pharmacological loss of CAIV in sperm from CAII knockout animals, show an even lower response to HCO_3_^−^. These results suggest that CAII and CAIV are required for optimal fertilization.

## Introduction

Post-testicular sperm undergo a multitude of maturation processes to acquire fertility in terms of penetrating the egg and generating a new unique individual. Sperm are transcriptionally and translationally silent. Therefore, the multiple physiological and biochemical modifications (1–5) must result from their interaction with the different environments through which they migrate (6–8). In the epididymis, including the caput, corpus, and cauda epididymides, a low bicarbonate (HCO_3_^−^) concentration and an acidic pH of the luminal fluid are important for sperm maturation, storage, and fertility (9–11). Segment-specific gene expression patterns of acid base transport proteins in epithelial cells (12–14) modulate the distinct fluid composition, which undergoes considerable changes along the epididymal duct (15). Several ion channels and transporters, such as cystic fibrosis transmembrane conductance regulator, SLC26A3/A6, and sodium bicarbonate exchanger, as well as V-ATPases have been proposed to produce the acidic and low HCO_3_^−^-concentrated fluid composition (7, 15–17). Carbonic anhydrases (CAs)^2^ have also been identified for HCO_3_^−^ resorption in the epididymis (18–21). Extracellular CA isoforms convert HCO_3_^−^ into CO_2_ which can then diffuse into the epididymal epithelial cells. Upon entry, CO_2_ can be reconverted into HCO_3_^−^ by intracellular CAs (22, 23). At the basolateral membrane, HCO_3_^−^ is then extruded by either AE2 and or sodium bicarbonate cotransporters (24, 25). Disturbance in the epididymal endothelium of the acid base homeostasis impairs sperm maturation processes and potentially causes male infertility (26).

Ejaculated sperm are not able to penetrate and fertilize the egg *in vivo*. They have to mature in the female genital tract. Chang (27) and Austin (28) discovered this essential process, termed capacitation, which includes strictly regulated and complex biochemical changes (4, 5). Female genital tract fluids are rich in HCO_3_^−^ and Ca^2+^ and exhibit an alkaline pH, supporting capacitation (8, 29). With regard to sperm motility, the capacitation process comprises major changes of the sperm beating pattern (30). Early HCO_3_^−^-mediated events (31) produce a fast, symmetrical flagellar beat and rapid progressive movement that allows sperm to travel the long distance through the uterus and the oviduct. Later events include the hyperactivated motility pattern characterized by high amplitude and asymmetrical flagellar beating, representing an increased torsional force (11, 32–34). Earlier work has proposed that hyperactivation may be the decisive factor in the release of sperm from the oviductal reservoir through detachment from the epithelium (35, 36). Our own findings indicate that sperm attachment and release may be independent of hyperactivation (37).

The pathway for HCO_3_^−^-evoked signaling in sperm is relatively well understood. This work focuses on the early HCO_3_^−^-induced signaling pathway. The downstream effects of HCO_3_^−^ have already been well described. Intracellular HCO_3_^−^ directly activates the soluble adenylyl cyclase, therefore increasing cAMP levels and activating PKA. This leads to increased protein tyrosine phosphorylation and an accelerated beat frequency (38–41). The mechanisms that initiate responses to HCO_3_^−^ in sperm have remained elusive. It has been proposed, but not shown, that the Na^+^/HCO_3_^−^ co-transporter NBC and the anion transporter SLC26A3/A6 are involved in the import of HCO_3_^−^ into sperm (42–44). It has also been suggested that CAs are involved in HCO_3_^−^ homeostasis because these enzymes catalyze the reversible reaction of CO_2_ to HCO_3_^−^ (45, 46). This work focuses on the earliest events in HCO_3_^−^ signaling in sperm.

So far, 16 CA isoforms have been identified (47), of which CAII and CAIV have been shown to be up-regulated during spermatogenesis (12, 48). CAIV is an extracellular glycosylphosphatidylinositol-anchored protein (49) whose involvement in the generation of HCO_3_^−^ in murine spermatozoa we have already established (50). That work showed that sperm of CAIV knockout mice display an HCO_3_^−^ disequilibrium at the cell surface and show a delayed and reduced increase of beat frequency in response to stimulation with HCO_3_^−^ and CO_2_. In this work, we determine the distribution and functional aspects of the CAII isoform as an intracellular counterpart to CAIV.

We show the presence of CAII in the epithelium lining, the epididymal duct, and the sperm tail. Specifically, we show that, in sperm lacking CAII, acceleration of the flagellar beat by HCO_3_^−^ and CO_2_ is delayed and reduced in magnitude. CAII and CAIV together contribute to almost 100% of total CA activity in sperm. CAII/CAIV double knockout mice display subfertility as well as reduced sperm motility. Our studies further reveal that, to uphold the HCO_3_^−^-induced rise in beat frequency, genetic double knockout sperm develop a compensatory mechanism. In conclusion, CAII and CAIV are key enzymes in the regulation of sperm motility and, therefore, essential for male fertility.

## Experimental Procedures

### 

#### 

##### Antibodies

Immunohistochemical and immunofluorescence detection of CAII and CAIV were performed with rabbit (rb) anti-CAII IgG (catalog no. sc25596, Santa Cruz Biotechnology, Heidelberg, Germany) and goat (gt) anti-CAIV IgG (catalog no. AF2414, R&D Systems, Wiesbaden, Germany). Capacitation was verified by anti-phosphotyrosine immunoblots (clone 4G10, catalog no. 05-321, Millipore, Schwalbach, Germany). Secondary HRP-conjugated donkey anti-gt IgG antibody was purchased from Santa Cruz Biotechnology (catalog no. sc2020), donkey anti-rb antibody from Abcam (catalog no. ab6802, Cambridge, UK) and goat anti-ms IgG antibody from Dianova (catalog no. 115-035-174, Hamburg, Germany). Biotinylated antibodies for DAB staining rabbit anti-gt (catalog no. BA-5000) and goat anti-rb (catalog no. BA-1000) were bought from Linaris (Dossenheim, Germany). Cy3-conjugated antibody was purchased from Acris (catalog no. R1435C3, San Diego, CA), and Alexa Fluor 488 was from Abcam (catalog no. ab150073).

##### Standard Solutions

Homogenization buffer for protein isolation contained 100 mm NaCl, 10 mm HEPES, 2 mm EDTA, 1 mm DTT, 2% Triton X-100, and protease inhibitor and was adjusted to pH 7.3 with 5% HCl solution. Lämmli buffer contained 0.3 m SDS, 0.15 m bromphenol, 20% glycerin, and 0.12 m tris(hydoxymethyl)aminomethane. 10× PBS washing buffer contained 14,00 mm NaCl, 26.8 mm KCl, 80 mm disodium hydrogen phosphate-dihydrate, and 14.7 mm KH_2_PO_4_, and washing buffer (TBS) contained 154 mm NaCl and 50 mm tris(hydroxymethyl)aminomethane. For PBS-T and TBS-T, 1% Tween® was added. Standard HEPES-buffered saline HS buffer (pH 7.4) contained 135 mm NaCl, 5 mm KCl, 2 mm CaCl_2_, 1 mm MgCl_2_, 20 mm HEPES, 5 mm glucose, 10 mm d,l-lactic acid, and 10 mm pyruvic acid. HSB buffer additionally contained 15 mm HCO_3_^−^. To induce capacitation, 5 mg/ml of fatty acid-free BSA was added to HSB buffer. High-potassium buffers K5.0, K7.0, and K9.9 for pH*_i_* calibration contained 5 mm NaCl, 135 mm KCl, 2 mm CaCl_2_, 1 mm MgCl_2_, 5 mm glucose, 10 mm d,l-lactic acid, 10 mm pyruvic acid, and either 20 mm MES (K5.0), HEPES (K7.0) or 3-{[2-hydroxy-1,1-bis(hydroxymethyl)ethyl]amino}-1-propanesulfonic acid (K9.0) and was adjusted to the indicated pH with 1 m NaOH or 5% HCl solution. All buffer ingredients were obtained from Sigma Chemical (Steinheim, Germany).

##### Animals, Phenotyping, and Fertility Analysis of CAII CAIV Double Knockout Mice

WT C57BL6/J and CAII knockout B6.D2-Car2^n^/J (CAII^−/−^) mice were obtained from The Jackson Laboratory (Bar Harbor, ME). CAIV knockout B6.129S1-Car4^tm1Sly/J^ (CAIV^−/−^) animals were provided by the laboratory of William S. Sly (Department of Biochemistry and Molecular Biology, St. Louis University School of Medicine, St. Louis, MO). Because of different chromosomal locations of the CAII (chromosome 8) and CAIV (chromosome 17) genes (51), CAII/CAIV double knockout (CAII^−/−^ CAIV^−/−^) animals were generated in accordance with approved protocols (no. 02/2011) by intercrossing individual heterozygous mice. According to Mendelian law, the probability of obtaining double knockout offspring is 6.25% at the F2 generation. For phenotype analysis of double knockout offspring, mutant mice were weighed once per week from day 21 on, body size was measured at the adult life stage, and organ weight of kidney and testis was determined and compared with WT mice. For further analysis, WT and double knockout testes were combined, embedded in paraffin, and used to study germ cell epithelia. For hematoxylin and eosin-stained testis, slices were examined with a bright-field microscope (Diaphot 300, Zeiss, Jena, Germany), and individual tubuli seminiferi contorti were documented. The thickness of germ cell epithelia was determined with Adobe Photoshop CS4 (Adobe Systems, San Jose, CA), whereby one tubule was calibrated orthogonally four times from the basal membrane to the tubule lumen, and advanced pixel lengths were converted into micrometer units. Results from three independently embedded testes for double knockout and WT mice with a total tubulus count ≥130 are shown as mean ± S.E.

The fertility of double knock-out mice was studied in long-term mating experiments. Double knockout mice were housed as individual mating pairs for 16 weeks. For comparison, other pairs included double knockout mice with a WT partner. The numbers and sizes of litters were recorded, and offspring per week of mating was calculated. Pure WT matings served as a control.

##### Sperm Preparation and Motility Analysis

Sperm were isolated from the cauda epididymidis and vasa deferentia after animals were sedated with isoflurane (Baxter, Unterschleißheim, Germany), followed by a cervical dislocation as described before (50). Sperm were allowed to swim out in HS buffer for 20 min at 37 °C and 5% CO_2_. Released sperm were washed twice with HS buffer (3 min at 300 × *g*) and resuspended in a final concentration of 1–2 × 10^7^ cells/ml in HS buffer. The sperm samples, washed and stored in HS, were used for all subsequent experiments. For further analysis of the effects of phospholipase C (PLC, Life Technologies) on sperm, 1 × 10^6^ cells/ml were incubated in 500 μl of HS buffer containing 2 units of PLC for 90 min at 37 °C in a shaking water bath. Sperm were sedimented (3 min at 300 × *g*) and resuspended in 250 μl of fresh HS buffer.

Computer-assisted sperm analysis (CASA) software (Medical Technology, Hamburg, Germany) reported average path velocity and straight line velocity (micrometer per second), percent motility, and sperm density (cells per milliliter). Sperm samples were mixed 1:1 with HS/5% BSA (VWR, Darmstadt, Germany) buffer and kept at 37 °C. All results are presented as mean ± S.E., calculated with SigmaPlot® v11.0 (Systat Software, Erkrath, Germany).

##### CA Activity in Sperm

CA enzyme activity experiments were performed on a quadrupole mass spectrometer (OmniStar GSD 320, Pfeiffer Vaccum, Asslar, Germany). Analysis was carried out as described previously (50). The loss of double-labeled ^13^C^18^O_2_ through several hydration and dehydration steps of CO_2_ and HCO_3_^−^ at 25 °C was measured over time by defining the mass number of initial (^13^C^18^O^18^O = 49 *m*/*z*), intermediate (^13^C^18^O^16^O = 47 *m*/*z*), and end products (^13^C^16^O^16^O = 45 *m*/*z*). OriginPro^TM^ 7 (OriginLab, Northampton, MA) was used to calculate the decay rate of the CA-catalyzed and -non-catalyzed reaction. Enzyme activity in units was calculated as defined by Badger and Price (74). From this definition, 1 unit corresponds to 100% stimulation of the noncatalyzed ^18^O depletion of doubly labeled ^13^C^18^O_2_. For the experiment, 6 ml of HS buffer was filled into a cuvette, and a non-catalyzed reaction was started by adding 6 μl of the double-labeled ^13^C^18^O_2_ for 8 min. 4 × 10^6^ sperm cells were added to measure the CA-catalyzed reaction for 10 min. Results are shown as mean ± S.E. of three independent experiments.

##### Immunoblotting

For CAII and CAIV protein detection, we prepared WT and knockout tissues from mice in the same way as described before (50). 60 μg of total protein and 30 μl of sperm sample were separated in a NuPAGE® 4–12% BisTris gel (Invitrogen) and blotted on nitrocellulose membranes (Invitrogen). Air-dried membranes were blocked with TBS/5% Slim Fast^TM^ for 1 h before primary antibody (rb anti-CAII IgG or/and gt anti-CAIV IgG, 1:1000, in 10% Roti Block (Roth, Karlsruhe, Germany)) incubation overnight at 4 °C. Membranes were washed twice and incubated first with HRP-conjugated anti-gt IgG (1:1000 in TBS-T) and then with HRP-conjugated anti-rb IgG (1:1000 in TBS-T), each for 1 h at room temperature. CAII and CAIV detection was carried out with ECL reagent (GE Healthcare) on a Chemie-Doc^TM^ XRS apparatus (Bio Rad).

To assess capacitation of sperm from WT and CAII^−/−^ CAIV^−/−^ mice, sperm from the cauda epididymidis and vasa deferentia were incubated for 180 min either in HS medium (37 °C, air atmosphere) or in capacitation medium (37 °C, 5% CO_2_). Protein extraction and blotting were carried out according to a protocol published previously (31). Membranes were incubated with anti-phosphotyrosine IgG (diluted 1:1000 in Roti-Block) overnight at 4 °C. After washing three times with TBS-T, the membranes were incubated with HRP-conjugated anti-mouse (diluted 1:10,000 in TBS-T) for 1 h at room temperature. Protein bands were detected with ECL reagent (GE Healthcare) on a Chemie-Doc^TM^ XRS apparatus (Bio Rad).

##### Immunohistochemistry

Organs were isolated from mice, fixed, and cut as described previously (50). Isolated sperm were air-dried and fixed in methanol for 15 min at room temperature.

Immunocytochemistry was performed as described previously (50). In brief, slices were blocked with PBS glucose oxidase buffer (10 mm glucose, 1 mm NaN_3_, and 0.4 units/ml glucose oxidase (Sigma)) and incubated with rb anti-CAII IgG (1:100 in PBS/5% BSA containing avidin 1:300) overnight at 4 °C, followed by incubation with biotinylated anti-rb IgG (1:200 in PBS/5% BSA containing biotin 1:50) for 30 min. The Vectastain® Elite ABC kit for peroxidase (Linaris, Dossenheim, Germany) was used, according to the protocol of the manufacturer, for signal enhancement. Detection was carried out with 3,3′diaminobenzidine (DAB, Sigma) as chromogen. For double immunostaining, slices were subsequently incubated with gt anti-CAIV IgG 1:100 in PBS/5% BSA overnight at 4 °C, followed by an incubation with biotinylated anti-gt IgG (1:200) in PBS/5% BSA. In this case, localization was determined with the Vectastain® ABC-AP kit (Linaris) using HistoRed as chromogen for alkaline phosphatase enzyme activity. Nuclear staining was performed with hematoxylin (Roth).

##### Double Immunofluorescence

After rehydration, sperm smears were incubated overnight at 4 °C with gt anti-CAIV IgG (1:100) in PBS/5% BSA. After two rinses in PBS, Cy3-conjugated anti-gt IgG (1:200) in PBS/5% BSA was incubated for 1 h at room temperature on the smears. To avoid possible cross-reactions, the smears were treated with 5% gt serum (Santa Cruz Biotechnology) in PBS for 45 min at room temperature. The following steps were done as described previously (53), with the following dilutions: rb anti-CAII IgG 1:100 in PBS/5% BSA, Alexa Fluor 488-conjugated anti-rb IgG (1:200) in PBS/5% BSA, DAPI 1:1000 in distilled water (Invitrogen). Image processing was carried out with Adobe Photoshop CS4 (Adobe Systems).

##### qRT-PCR

Tissue homogenization, RNA isolation, and cDNA synthesis were performed as described previously (49). cDNA served as a template for the following RT-PCR, and RNA expression of CAII, CAIV, and CAXIV in tissues from the male reproductive tract was assessed by relative quantification with the ΔΔCt method (54). 18S rRNA served as the endogenous standard and kidney as the reference tissue. Amplification and detection were carried out according to an Applied Biosystems protocol, each cDNA template as triplicate with a respective TaqMan® gene expression assay (Applied Biosystems, Darmstadt, Germany) on a StepOnePlus^TM^ cycler and software (Applied Biosystems). Results are presented as mean real-time quantitative values or relative amounts ± S.E., each from three independent experiments.

##### Determination of Flagellar Beat Frequency

Beat frequency was determined as described previously (50). In brief, flagellar beat frequency was observed on an inverted microscope (Diaphot 300, Nikon, Tokyo, Japan) and recorded at 300 Hz in a 1200 × 1400 pixel region with an IDT M3 high-speed camera (IDT Inc., Tallahassee, FL) and Motion Studio 64 software (Imaging Solutions, Regensburg, Germany). Determination of single sperm beat frequency was performed as described previously (55). Single sperm sequences were cut, arranged, and contrasted by ImageJ v1.37 software. Images of maximum amplitudes were merged into one sum file with MetaMorph v7.1 (Molecular Devices, Sunnyvale, CA) and analyzed by a semiautomated algorithm written in Igor Pro^TM^ v6.04 (Wavemetrics, Lake Oswego, OR). Data are shown as mean ± S.E., calculated with SigmaPlot® v11.0 (Systat Software) and with a minimum of 17 single sperm for each waveform experiment (for exact sperm numbers, see the figure legends).

##### Dye Loading and pH_i_ Measurements

To measure the steady-state pH*_i_*, sperm were loaded and measured as described previously (53). In brief, 250 μl of HS buffer was spiked with 0.1 μm Pluronic®-Fl27 (Invitrogen) and 0.1 μm pH-sensitive 2′,7′-bis-(2-carboxyethyl)-5-(and-6)-carboxyfluorescein, acetoxymethyl ester (Invitrogen) and mixed with 250 μl of HS-stored sperm (3 × 10^6^ cells/ml) suspension. After three washing steps, cells were measured on a Nikon Eclipse TE2000-U microscope equipped with a monochromator (Till Photonics, Munich, Germany). An intracellular calibration was performed by suspension of the dye-loaded sperm in K^+^-based medium variously buffered at pH 5.0, 7.0, or 9.0 and treatment with the K^+^-selective ionophore nigericin (Sigma Chemicals). For pH*_i_* equilibration, the fluorescence ratio of 436/488 was transferred to a cell-specific pH*_i_* (56). To measure the kinetics of changes in the pH*_i_* dye loading, the experimental procedures were carried out the same way as described above, with the following exceptions. 250 μl of the sperm solution (3 × 10^6^ cells/ml) was mixed with an equal volume of HS buffer containing 0.1% PowerLoad^TM^ and 0.5 μm pHrhodo^TM^ Red acetoxymethyl ester (Invitrogen). Cells were incubated for 30 min in the dark at room temperature, washed two times with fresh HS buffer, and subsequently used to measure the pH*_i_*. Fluorescence was sampled during 50 ms of excitation, applied at 1 Hz. Changes in fluorescence were normalized to resting fluorescence (F/F0) with SigmaPlot® 11.0 (Systat Software).

## Results

### 

#### 

##### CAII and CAIV Distribution in the Male Reproductive Tract and in Sperm

**FIGURE 1.**
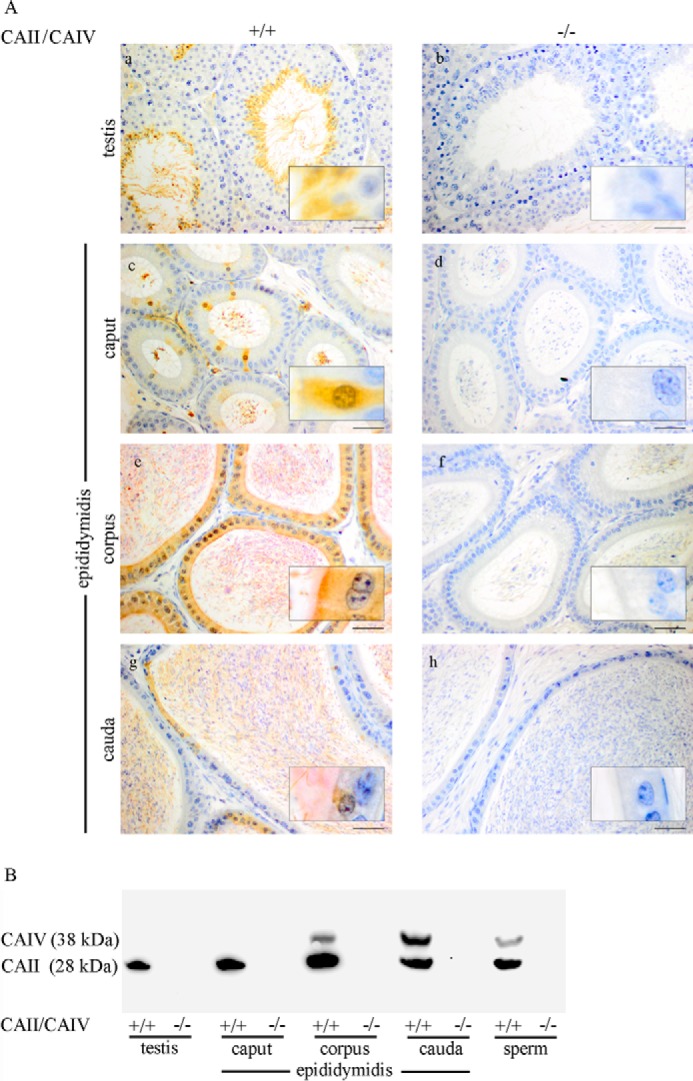
**Distribution of CAII and CAIV in the male reproductive tract.** *A* and *B*, double immunohistochemical staining (*A*) and immunoblots (*B*) were performed with WT (+/+) and CAII^−/−^ CAIV^−/−^ (−/−) samples. Tissue slices were stained with DAB for CAII (*brown*) and HistoRed for CAIV (*red*) signals. CAII is present in elongated spermatids, epididymal spermatozoa, single epithelial cells of the caput and the cauda epididymidis, and nearly all epithelial cells of the corpus epididymidis. CAIV is localized in the stereocilia network of the corpus and the cauda epididymidis as well as in luminal sperm after passing the corpus region. In Western blot analyses, CAII (28 kDa) is detectable in the testis, all parts of the epididymidis, and cauda sperm. A specific CAIV band at 38 kDa is only present in the corpus and the cauda epididymidis as well as in sperm. Neither CAII nor CAIV are detectable in any of the double knockout tissues or in the protein samples, which served as control (*Scale bars* = 50 μm (*a–h*) and 10 μm (*insets*).

**FIGURE 2.**
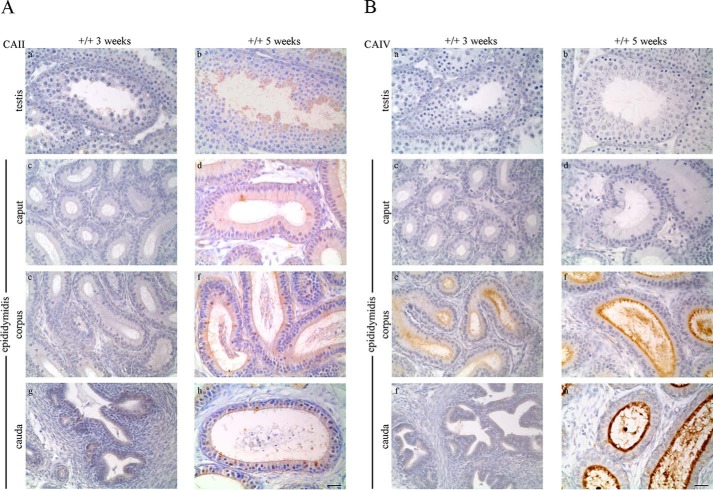
**CAII and CAIV protein detection changes with puberty in the male reproductive tract.** *A*, CAII protein detection by immunohistochemical staining appears with the onset of puberty (5 weeks) in elongated spermatids, single epithelial cells, and the stereocilia network of the caput, corpus, and cauda epididymidis. *B*, CAIV immunoreactivity is localized in 3-week-old mice only in the stereocilia network of the corpus epididymidis but not detectable in the testis, caput, and cauda epididymidis. In tissues from pubescent mice, CAIV is present in the corpus and cauda epididymidis (*Scale bars* = 25 μm).

Double immunostaining (Fig. 1*A*) was performed to localize CAII and CAIV in the testis, epididymis, and sperm. CAII was detected with DAB (brown) and CAIV with Texas Red (red). WT testis (Fig. 1*A*, *a*) shows a specific CAII signal in elongated sperm, whereas early germ cell states are CAII-negative. In the epididymis, CAII is present in single epithelial cells of the caput (Fig. 1*A*, *c*), the cauda epididymidis (Fig. 1*A*, *g*), and in nearly all cells of the corpus epididymidis (Fig. 1*A*, *e*). CAIV signals are not detectable in WT testis (Fig 1*A*, *a*) and the caput epididymidis (Fig. 1*A*, *c*). However, a specific immunoreaction is visible in the apically located stereocilia network of epithelial cells in the corpus epididymidis (Fig. 1*A*, *e*) as well as in the cauda epididymidis (Fig. 1*A*, *g*). Luminally located sperm show a specific CAII signal in the testis and in all parts of the epididymis. In contrast to CAII, CAIV is not detectable in luminal sperm of the testis and caput epididymidis. CAIV is only present in sperm from the corpus and cauda epididymidis. Tissue from CAII^−/−^ CAIV^−/−^ mice, which served as a negative control, does not show any specific CAII and CAIV immunoreaction (Fig. 1*A*, *b*, *d*, *f*, and *h*). The same results were obtained from Western blot analyses, as shown in Fig. 1*B*. Protein extracts from WT and CAII^−/−^ CAIV^−/−^ mice were stained for the presence of CAII and CAIV. In the WT testis and caput epididymidis, only a CAII signal (28 kDa) is detectable, whereas the WT corpus and cauda epididymidis display signals for CAII and CAIV (38 kDa). Protein extracts from isolated WT sperm show a prominent immunoreactive CAII and a weaker CAIV band. No signal was detected in any tissue or sperm of CAII^−/−^ CAIV^−/−^ animals (Fig. 1*B*, −/−). To analyze development-dependent protein expression in the male genital tract, we stained WT tissues (+/+) of prepubertal (3-week-old) and pubescent (5-week-old) mice with antibodies against CAII (Fig. 2*A*) and CAIV (Fig. 2*B*). CAII is not detectable in 3-week-old WT testis (Fig. 2*A*, *a*) and the distinct parts of the epididymis (Fig. 2*A*, *c*, *e*, and *g*). Puberty leads to significant changes in CAII distribution. In tissues from 5-week-old animals, CAII is localized throughout the entire genital tract with similar but weaker signals (Fig. 2*A*, *b*, *d*, *f*, and *h*) compared with the tissues from adult animals in Fig. 1*A*. In contrast to CAII, a specific CAIV signal is already present in prepubertal (3-week-old) WT tissue from the corpus epididymidis (Fig. 2*B*, *e*). No CAIV signal is present in the 5-week-old testis (Fig. 2*B*, *b*) and caput (Fig. 2*B*, *d*). Intense staining occurs in the apically located stereocilia network of the corpus epididymidis (Fig. 2*B*, *f*) and the cauda epididymidis (Fig. 2*B*, *h*) of 5-week-old mice.

**FIGURE 3.**
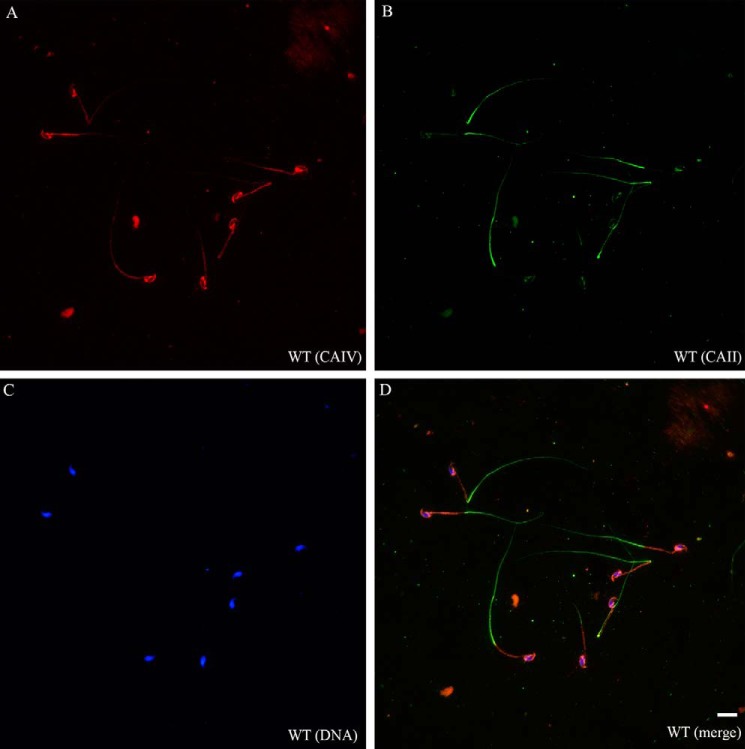
**CAII and CAIV have distinct localizations in murine spermatozoa.** *A–D*, double immunofluorescence on fixed WT sperm indicates a CAII signal (*green*) in the principal piece of the sperm tail, whereas CAIV (*red*) is present in the acrosome and the plasma membrane of the entire sperm tail, predominantly in the mid-piece. CAII^−/−^ CAIV^−/−^ sperm did not show any signal (data not shown). Nuclei were stained with DAPI. *Scale bar* = 10 μm.

To localize CAII and CAIV more systematically in epididymal sperm, we performed double immunofluorescence staining (Fig. 3). CAII signals (green) are detectable in the cytoplasm of the principal piece of sperm tail. CAIV signals (red) are localized in parts of the acrosome and in the plasma membrane of the entire sperm tail, predominantly in the mid-piece. In comparison with WT sperm, sperm from double knockout mice are negative for CAII and CAIV (data not shown).

##### CAII and CAIV Are the Most Abundant Isoforms in Sperm

**FIGURE 4.**
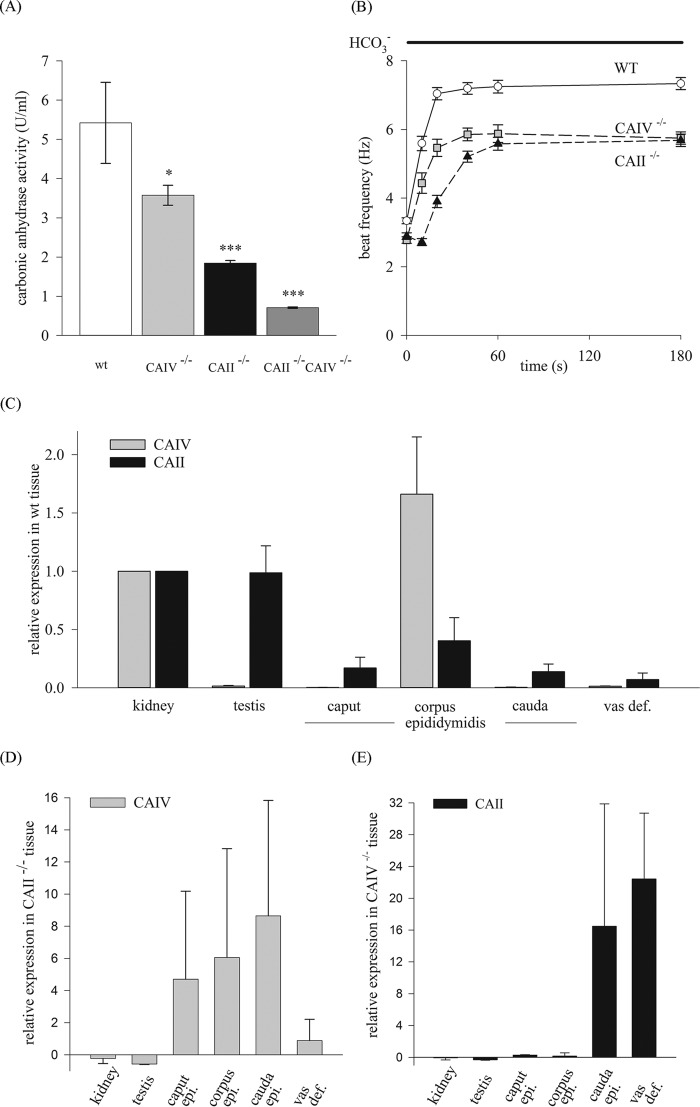
**CAII and CAIV activity in sperm and expression levels in the male reproductive tract of WT, CAII^−/−^, and CAIV^−/−^ mice.** *A*, CA activity was measured with mass spectrometry. In comparison with WT sperm, the deletion of CAII or CAIV leads to a reduction in activity of 32% and 65%, respectively. CA activity in CAII^−/−^ CAIV^−/−^ mice is 0.7 units/ml (± 0.02), which equals a reduction of 86.9%. Data are mean ± S.E. of six or more measurements from three independent experiments. The *asterisks* refer to the values of the bar for WT. *, *p* ≤ 0.05; **, *p* ≤ 0.01; ***, *p* ≤ 0.001. *B*, analysis of the single-sperm beat frequency response to 15 mm HCO_3_^−^. CAII^−/−^ and CAIV^−/−^ sperm display a delayed and reduced acceleration of beat frequency. Data are mean ± S.E. of ≥40 single sperm. *C*, expression of CAII and CAIV mRNA in WT tissue detected by qRT-PCR. *D* and *E*, in relation to WT tissue, CAIV is overexpressed in the caput, corpus, and cauda epididymidis (*epi.*) of CAII^−/−^ mice (*D*), whereas CAII expression is highly increased in the cauda epididymidis and vas deferens (*vas def.*) of CAIV^−/−^ animals (*E*) (*n* = 3).

To determine CAII and CAIV enzyme activity, mass spectrometry was performed. A comparison of total CA activity between the sperm of WT and CAII or CAIV knockout animals provides information about the relative activity of these CA isoforms in sperm. The results illustrated in Fig. 4*A* indicate a total CA enzyme activity in WT sperm of 5.20 ± 0.2 units/ml. In comparison with WT sperm, CAIV^−/−^ leads to a significant reduction of 31.4% (3.57 ± 0.25 units/ml) and CAII^−/−^ of 63.2% (1.84 ± 0.07 units/ml). We consequently measured the CA activity in sperm of CAII^−/−^ CAIV^−/−^ animals. The detected activity of 0.7 units/ml (± 0.02) equals a reduction of 86.9% compared with WT sperm. This activity is less than the activity measured in native *Xenopus* oocytes (1.5 units/ml), which do not express CA at all (57) Because CAs are involved in generating HCO_3_^−^, we examined the influence of CAII and CAIV on the HCO_3_^−^-mediated early activation of sperm. For this, we determined the beat frequency of single sperm as a function of time by application of 15 mm HCO_3_^−^ (Fig. 4*B*). WT sperm respond to HCO_3_^−^ with an acceleration of their beat frequency from 3.3 ± 0.09 Hz to a maximum of 7.04 ± 0.18 Hz within the first 20 s. Sperm from CAIV, as well as from CAII knockout mice, show a delayed and reduced HCO_3_^−^ response compared with WT sperm. The sperm beat frequency of CAIV^−/−^ animals rises from 2.78 ± 0.09 Hz to 5.47 ± 0.25 Hz within 20 s and displays a maximum of 5.88 ± 0.26 Hz (t = 60 s). Sperm from CAII^−/−^ animals accelerate from 2.89 ± 0.1 Hz to 3.9 ± 0.18 Hz in the first 20 s, reaching a maximum of 5.58 ± 0.19 Hz after 60 s of stimulation. Similar beat frequency results were obtained by stimulating sperm with 2% CO_2_ (Fig. 8*B*). These findings confirm the direct involvement of both enzymes in the HCO_3_^−^-mediated pathway.

##### Compensatory Expression of CAII and CAIV in Tissues of Single Knockout Mice

qRT-PCR analyses were performed to examine the expression of CAII and CAIV mRNA in tissues of the male reproductive tract. First, the expression in WT tissue was determined (Fig. 4*C*). In relation to the kidney, which served as the internal standard (relative expression value, 1.0), CAIV mRNA is only expressed in the corpus epididymidis (1.66 ± 0.49). CAII mRNA is present in all parts of the reproductive tract, with the highest expression values of 0.99 ± 0.23 in the testis. Next, CAIV mRNA expression was determined in CAII^−/−^ animals and vice versa to verify a potential compensatory overexpression. Fig. 4*D* shows the relative CAIV expression in CAII^−/−^ knockout animals. In these experiments, WT tissue served as internal standards. An overexpression of CAIV in CAII knockout tissues can be detected in the caput (5.70 ± 5.48), corpus (7.06 ± 6.78), cauda epididymidis (9.65 ± 7.18), and vas deferens (1.89 ± 1.32). In contrast, CAIV^−/−^ animals show (Fig. 4*E*) a considerable overexpression of CAII in the cauda epididymidis (17.48 ± 15.4) and vas deferens (23.42 ± 8.3).

##### Unaltered Characteristics in the Testis and Sperm of CAII/CAIV Double Knockout Mice

**FIGURE 5.**
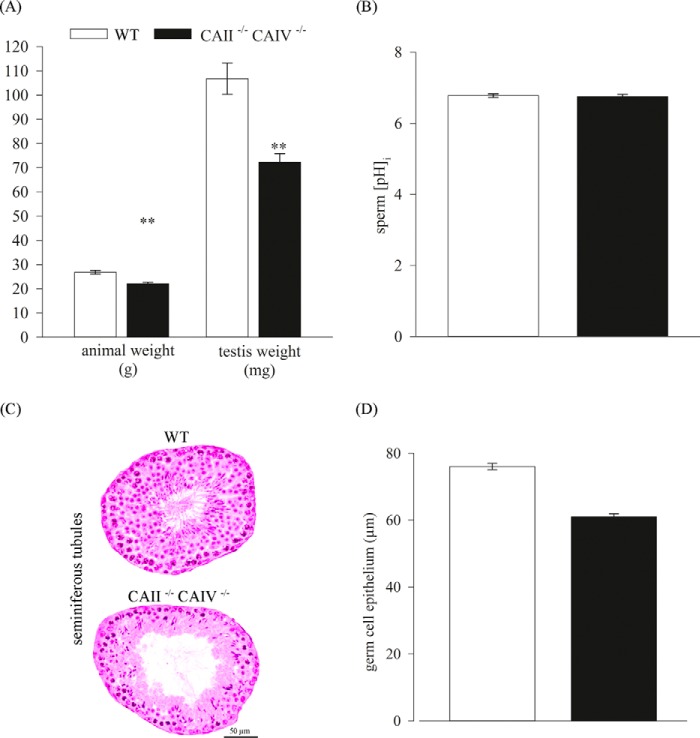
**Phenotype of CAII^−/−^ CAIV^−/−^ offspring.** *A*, adult CAII^−/−^ CAIV^−/−^ mice show a significant reduction in body and testis weight in comparison with WT mice (*n* = 4). The *asterisks* refer to the values for animal and testis weight of the bar for WT. **, *p* ≤ 0.01. *B*, no change is detectable in the steady-state pH*_i_* of isolated sperm from WT and double knockout animals (*n* ≥ 12). *C*, cross-sections through WT (*top*) and CAII^−/−^ CAIV^−/−^ (*bottom*) testis indicate a thinner germ epithelium in double knockout testis. Representative sections are shown. *D*, the analysis of 130 tubules of three different WT and double knockout testes displays a reduction of 20% of the germ cell epithelium in CAII^−/−^ CAIV^−/−^ mice.

**FIGURE 6.**
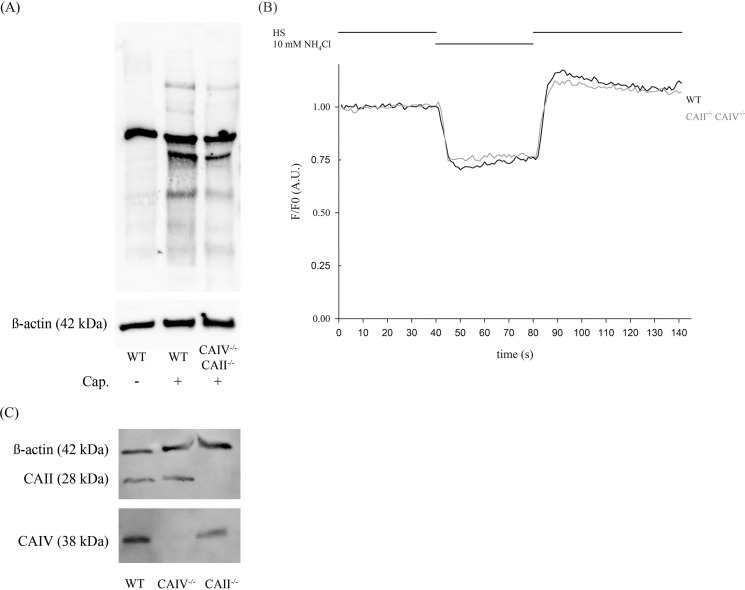
**Basal pH level and ability to capacitate of CAII^−/−^ CAIV^−/−^ sperm is not altered.** *A*, immunoblots were performed with protein extracts from untreated or capacitated sperm from WT (+/+) and CAII^−/−^ CAIV^−/−^ (−/−) mice. CAII^−/−^CAIV^−/−^ sperm do not show any changes in the protein phosphorylation pattern induced by capacitation (*Cap*) compared with WT sperm. *B*, CAII^−/−^ CAIV^−/−^ sperm do not show any defects in intracellular alkalization upon stimulation with ammonium chloride. Sperm of both WT (*black trace*) and CAII^−/−^ CAIV^−/−^ (*gray trace*) mice were perfused continuously with HS buffer and 10 mm ammonium chloride as indicated. Shown are averaged normalized traces of 34 WT and 39 CAII^−/−^ CAIV^−/−^ sperm of two animals, respectively. *A.U.*, arbitrary units. *C*, CAII^−/−^ sperm show a reduced CAIV protein level, whereas the amount of CAII protein in sperm from CAIV^−/−^ mice corresponds to that of WT mice.

Double knockout mice were bred to study the role of CAII and CAIV *in vivo*. Double knockout animals were generated by mating individual heterozygous mice. CAII^−/−^ CAIV^−/−^ offspring are viable and do not display any visible differences in size compared with WT animals at birth. Also, macroscopic studies of the kidney and the male reproductive tract do not show any difference compared with WT organs (data not shown). However, a closer examination after weaning reveals a significant reduction in animal and testis weights compared with WT mice. The mutant offspring was weighed once a week from day 21 on, and a reduced weight in all life stages was detected. Fig. 5*A* shows the body weight of 10-week-old male mice. CAII^−/−^ CAIV^−/−^ mice have an average weight of 22.13 ± 0.61 g, whereas WT mice of the same age weigh 26.82 ± 0.78 g. Adult testes of CAII^−/−^ CAIV^−/−^ mice reach an average weight of 72.33 ± 3.51 mg, which is a reduction of 32.2% compared with WT testes (106.75 ± 6.5 mg) (Fig. 5*A*). Because CAs are essential for the regulation of pH*_i_*, we determined the steady-state pH*_i_* of the sperm of double knockout mice (Fig. 5*B*). Mean values of pH*_i_* in CAII^−/−^ CAIV^−/−^ cells (pH*_i_* = 6.75 ± 0.07) do not show any significant change compared with the sperm of WT animals (pH*_i_* = 6.78 ± 0.05). Through morphological studies of the testes (Fig. 5*C*), we observed a thinner germ epithelium in the seminiferous tubules, which might be an explanation for the significant lower testis weight. By measuring 130 tubuli of three different mice, an average germ cell epithelium of 60.97 ± 0.93 μm in CAII^−/−^ CAIV^−/−^ animals and 76.00 ± 0.95 μm in WT testes was detected (Fig. 5*D*). We used capacitating conditions to compare the sperm of WT and CAII^−/−^ CAIV^−/−^ mice. Sperm of both animals were able to show tyrosin phosphorylation as an indicator of late effects of bicarbonate (Fig. 6*A*). Because no differences in the steady-state pH*_i_* of WT and double knockout sperm were observed, we next investigated whether there was a difference in the kinetics of intracellular alkalization upon stimulation with ammonium chloride. Fig. 6*B* shows the response of WT sperm (*black line*) and CAII^−/−^ CAIV^−/−^ sperm (*gray line*) to ammonium chloride. The decrease of fluorescence is 25.5% in WT and 22.6% in CAII^−/−^ CAIV^−/−^ sperm. To verify the compensatory expression of CAII in CAIV^−/−^ mice and vice versa, we used Western blot analysis. Sperm of CAII^−/−^ animals show a reduced CAIV protein level whereas the CAII protein level in sperm of CAIV^−/−^ mice corresponds to that of WT mice (Fig. 6*C*).

##### CAII/CAIV Deletion Drastically Affects Sperm Motility and Fertility

**FIGURE 7.**
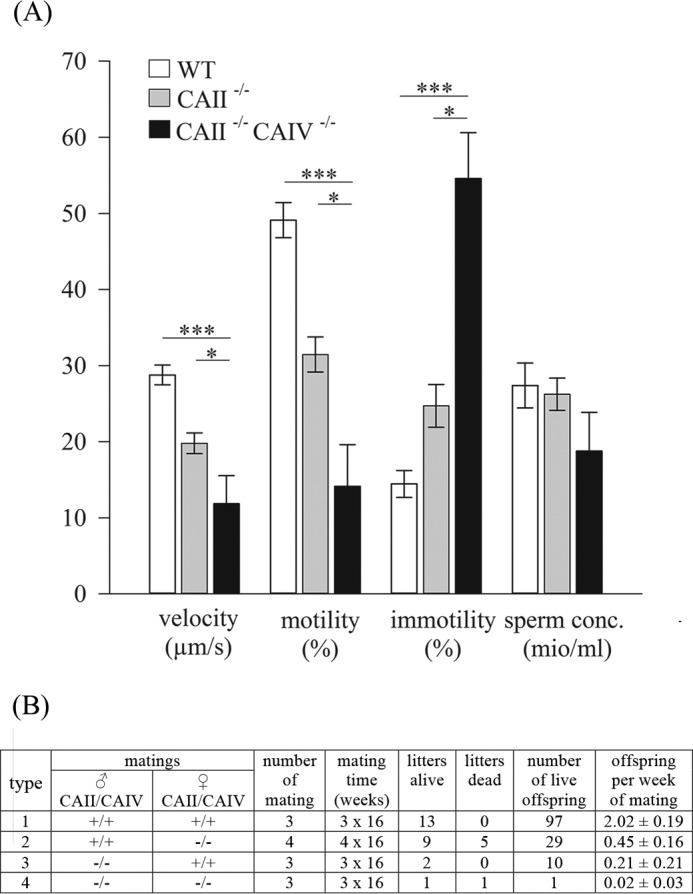
**CAII^−/−^ CAIV^−/−^ mice are subfertile and display reduced sperm motility.** *A*, CASA analyses were performed to characterize the sperm population of WT, CAII^−/−^, and CAII^−/−^ CAIV^−/−^ mice. Velocity and motility are reduced in CAII^−/−^ and in CAII^−/−^ CAIV^−/−^ sperm, whereas the percentage of immotile sperm is increased significantly. Sperm concentrations (*sperm conc.*) are not affected. Data are mean ± S.E. of seven or more measurements from three independent experiments. *, *p* ≤ 0.05; ***, *p* ≤ 0.001. *B*, offspring analysis from mating pairs indicated as type 1, 2, 3, and 4. The offspring per week of mating were calculated from total mating time and number of live offspring. Both CAII/CAIV double knockout sexes indicate a significant reduced offspring per week.

To assess different motility patterns, we performed CASA analyses with sperm populations from CAII^−/−^ and CAII^−/−^ CAIV^−/−^ animals (Fig. 7*A*). In comparison with WT, sperm of CAII^−/−^ and CAII^−/−^ CAIV^−/−^ animals indicate a reduced velocity and motility. The mean VSL reported by CASA are 28.77 ± 1.29 μm/s (WT), 19.79 ± 1.36 μm/s (CAII^−/−^), and 11.84 ± 3.72 μm/s (CAII^−/−^ CAIV^−/−^). The percentage of motile sperm is 49.11 ± 2.31% (WT), 31.45 ± 2.30% (CAII^−/−^), and 14.14 ± 5.46% (CAII^−/−^ CAIV^−/−^). In contrast, the mean percentage of immotile sperm rises from 14.45% ± 1.78% in WT to 24.70% ± 2.81% in CAII^−/−^ to 54.57% ± 6.03% in CAII^−/−^ CAIV^−/−^ mice. No significant changes can be detected in sperm concentrations between the different genotypes (WT, 27.38 ± 2.95 million/ml; CAII^−/−^, 26.23 ± 2.11 mio/ml; CAII^−/−^ CAIV^−/−^, 18.76 ± 5.08 mio/ml). Fertility was determined by offspring analysis from three independent mating experiments. As indicated in Fig. 7*B*, WT males were mated with WT females (type 1) or CAII^−/−^ CAIV^−/−^ females (type 2). Furthermore, WT females were mated with CAII^−/−^ CAIV^−/−^ males (type 3). Type 4 indicates the mating of CAII^−/−^ CAIV^−/−^ males with CAII^−/−^CAIV^−/−^ females. Each pair type was mated over a period of 16 weeks, and the numbers of litters and pups were recorded. WT mating (type 1) produced an average offspring per week of 2.02 ± 0.19. Smaller litter sizes resulted from matings of CAII^−/−^ CAIV^−/−^ females with WT males (type 2). In this case, four individual matings produced nine living litters with a total number of offspring of 29, which equals 0.45 ± 0.16 pups and five still births. The intercrossing of CAII^−/−^ CAIV^−/−^ males with WT females (type 3) resulted in only two litters with 10 pups from three independent matings. Consequently, the calculated offspring per week of mating was 0.21 ± 0.21 and indicates a reduced fertility of 90% for double mutant male mice. Mating of CAII^−/−^ CAIV^−/−^ females with CAII^−/−^ CAIV^−/−^ males (type 4) increases the subfertility of the animals. The breeding resulted in only two litters, one alive and one dead. The calculated offspring per week of 0.02 ± 0.21 points to near infertility of this kind of breeding. These results indicate that the fertility of both sexes is reduced dramatically because of loss of CAII and CAIV.

##### Compensatory Mechanisms in the Sperm of Genetic CAII^−/−^ CAIV^−/−^ Mice Uphold the Essential HCO_3_^−^-induced Pathway

**FIGURE 8.**
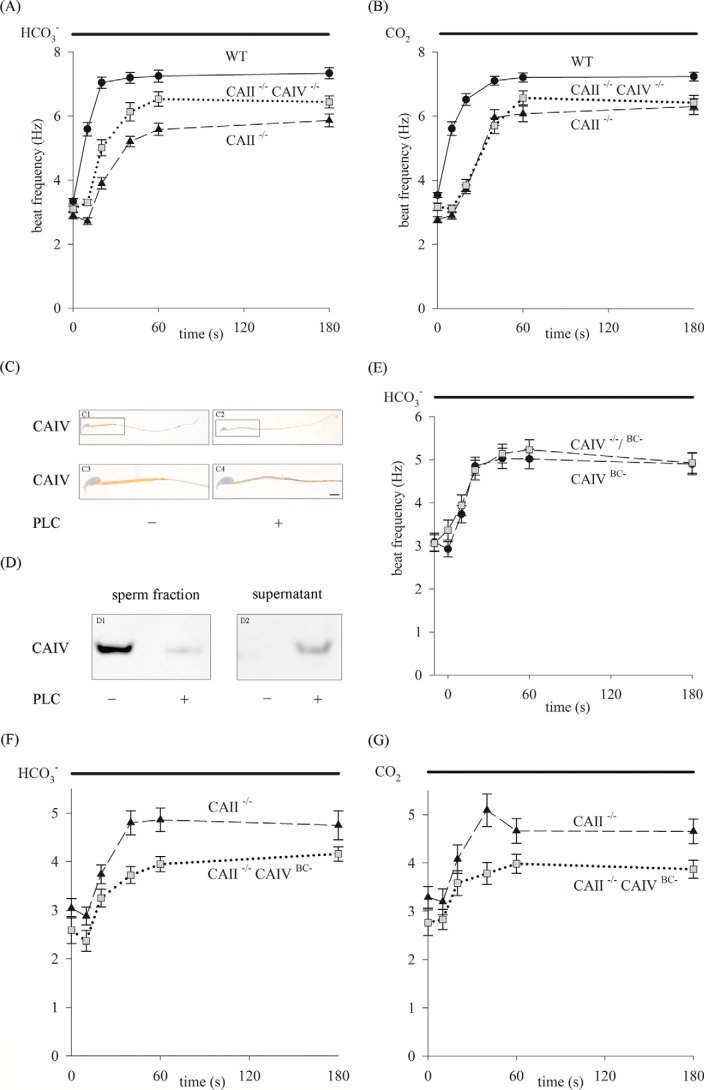
**Analysis of sperm beat frequency on a single-cell level.** *A* and *B*, sperm of WT, single CAII^−/−^, and CAII^−/−^ CAIV^−/−^ animals were stimulated with 15 mm HCO_3_^−^ (*A*) or 2% CO_2_ (*B*). With both protocols, CAII^−/−^ sperm display the strongest delayed and reduced beat frequency increase compared with CAII^−/−^ CAIV^−/−^ and WT sperm. *C* and *D*, biochemical CAIV protein elimination by PLC treatment of WT sperm was tested successfully by DAB staining (*C*, *scale bar* = 4 μm) and Western blot analysis (*D*). *E*, in single-cell analysis, PLC-treated WT (*CAIV^BC^*^−^) and genetic CAIV^−/−^ sperm (*CAIV*^−/−/^*^BC^*^−^) display the same response upon HCO_3_^−^ stimulation. *F* and *G*, shown is the response of genetic CAII^−/−^ and CAII^−/−^ CAIV^BC−^ double knockout sperm to HCO_3_^−^ (*F*) and CO_2_ (*G*). The beat frequency of CAII^−/−^ CAIV^BC−^ sperm increases only to a maximum of 4.16 ± 0.15 Hz with HCO_3_^−^ and of 3.87 ± 0.19 Hz with CO_2_ application (t = 80 s) and displays the weakest response. Data are mean ± S.E. of ≥17 single sperm.

To examine to what extent the deletion of CAII and CAIV affects the HCO_3_^−^-mediated pathway in early sperm activation, we performed waveform analyses with single sperm. Fig. 8, *A* and *B*, shows the beat frequency of sperm from WT (*solid line*), CAII^−/−^ (*dashed line*), and CAII^−/−^ CAIV^−/−^ (*dotted line*) mice over time by perfusion with HCO_3_^−^ (Fig. 8*A*) and CO_2_ (Fig. 8*B*). The WT sperm beat frequency accelerates within 20 s from 3.34 ± 0.09 Hz to 7.04 ± 0.18 Hz with HCO_3_^−^ and from 3.53 ± 0.08 Hz to 6.51 ± 0.19 Hz with CO_2_. CAII^−/−^ sperm show an increase in beat frequency within the first 20 s from 2.90 ± 0.10 Hz to 3.90 ± 0.18 Hz by HCO_3_^−^ and from 2.77 ± 0.09 Hz to 3.72 ± 0.14 Hz by CO_2_ application. The respective maximum of 5.86 ± 0.20 Hz (HCO_3_^−^) and 6.30 ± 0.25 Hz (CO_2_) is reached after 180 s of stimulation. Sperm of CAII^−/−^ CAIV^−/−^ mice increase their beat frequency from 3.10 ± 0.08 Hz to 5.01 ± 0.25 Hz in the first 20 s by HCO_3_^−^ and from 3.18 ± 0.11 Hz to 3.84 ± 0.19 Hz by CO_2_ perfusion and reach a maximum after 60 s of 6.44 ± 0.19 Hz (HCO_3_^−^) and 6.57 ± 0.21 (CO_2_). These results reveal that the additional CAIV gene loss of CAII^−/−^ mice does not potentiate the adverse effects of the mutant. A residual CA activity in the double KO sperm preparations could be due to contamination by somatic cells.

To circumvent any genetic compensatory mechanisms, we aimed to generate biochemical CAII/CAIV double knockout sperm by applying the enzyme PLC to sperm from CAII^−/−^animals. PLC cleaves off glycosylphosphatidylinositol-anchored proteins such as CAIV, yielding biochemically induced double knockout sperm (CAII^−/−^ CAIV^BC−^) (58).

To confirm the cleavage of CAIV, WT sperm were first treated with PLC to produce biochemical CAIV knockout sperm (CAIV^BC−^). These PLC-incubated sperm show a reduction of the immunoreaction with the CAIV antibody in the entire sperm tail compared with WT sperm (Fig. 8*C*). The same results can be obtained by Western blot analysis (Fig. 8*D*). The PLC-treated sperm fraction displays a diminished band intensity in comparison with non-treated WT sperm (Fig. 8*D*, *left panel*). Additionally, the CAIV protein is present in the corresponding supernatant of PLC-incubated sperm, whereas no CAIV-specific band is detectable in the supernatant of non-treated WT sperm (Fig. 8*D*, *right panel*). To exclude that the treatment with PLC has an effect on the HCO_3_^−^-mediated beat frequency acceleration, we incubated sperm from genetic CAIV^−/−^ mice with PLC (CAIV^−/−^/^BC−^). The response of such cells to HCO_3_^−^ compared with CAIV^BC−^ sperm is not significantly different (Fig. 8*E*). The beat frequency of CAIV^−/−^/^BC−^sperm increases during 180 s from 3.06 ± 0.19 Hz to 4.93 ± 0.24 Hz after stimulation with HCO_3_^−^. Similar values were obtained with CAIV^BC−^ sperm (3.09 ± 0.21 Hz to 4.90 ± 0.25 Hz). The fact that sperm of CAIV^−/−^/^BC−^ mice respond similar to HCO_3_^−^ as CAIV^BC−^ cells confirms that treatment with PLC does not affect the response to HCO_3_^−^ in early sperm activation because of a loss of other glycosylphosphatidylinositol-anchored proteins.

In the next step, sperm from CAII^−/−^ mice were treated with PLC, creating CAII^−/−^ CAIV^BC−^ sperm. These cells are genetically deficient for CAII and biochemically deficient for CAIV. The beat frequency of CAII^−/−^ CAIV^BC−^ sperm was analyzed during stimulation with HCO_3_^−^ and CO_2_ and compared with sperm from CAII^−/−^ animals. Fig. 8, *F* and *G*, illustrates the results from single-cell beat frequency measurements over time. The weakest response to HCO_3_^−^ could be detected in CAII^−/−^ CAIV^BC−^ sperm. The beat frequency increases from 2.59 ± 0.28 Hz to 3.95 ± 0.16 Hz (t = 60 s) with HCO_3_^−^ application, whereas the frequency of CAII^−/−^ sperm rises from 3.04 ± 0.20 Hz to 4.86 ± 0.24 Hz after 60-s stimulation with HCO_3_^−^. Similar results are obtained when stimulating the cells with CO_2_ (CAII^−/−^, 3.29 ± 0.23 Hz to 4.66 ± 0.25 Hz, t = 60 s; CAII^−/−^ CAIV^BC−^, 2.76 ± 0.26 Hz to 3.98 ± 0.19 Hz, t = 60 s). In conclusion, CAII^−/−^ CAIV^BC−^ sperm show the lowest response in beat frequency upon stimulation with HCO_3_^−^ or CO_2_. However, sperm from CAII^−/−^ CAIV^−/−^ animals respond with a higher beat frequency to both stimuli (Fig. 8, *A* and *B*).

## Discussion

### 

#### 

##### Loss of CAII and CAIV Substantially Affects Sperm Motility and Murine Fertility

Our immunohistochemical and PCR studies show the presence of CAII in epithelial cells of all parts of the epididymis. These results concur with studies in rats (19, 59) and humans (15). Because of its enzymatic activity, CAII is presumed to be involved in the acidification process of the epididymal fluid. The strongest CAII protein signal was detected in epithelial cells of the corpus epididymidis. This result indicates a dominant regulatory function of CAII in this section, where pH-dependent sperm maturation processes like protein transfer, lipid remodeling, and protein modifications take place (1, 60–63). Furthermore, sperm storage in a quiescent state in the cauda epididymidis is a pH- and HCO_3_^−^-dependent process in which CAII is likely to be involved (64).

In contrast to CAII, this study and a previous study (50) demonstrate the presence of the membrane-bound CAIV only in the stereocilia network of the corpus epididymidis. Here, the transfer of CAIV from the stereocilia of the epithelial cells to the sperm plasma membrane takes place (50, 65), which may explain the exclusive and high expression levels in this part of the epididymis. The high CAIV protein content in the cauda epididymidis we detected with Western blot analysis is the result of the luminal CAIV-positive sperm, which were not flushed out prior to protein isolation. An additional function of CAIV, together with the co-localized CAII in corpus epithelial cells, could be HCO_3_^−^ resorption combined with parallel intracellular H^+^ generation (22, 66). This hypothesis is supported by the work of Au and Wong (20), who showed that the intravenous application of the nonspecific CA inhibitor acetazolamide blocks luminal acidification in the rat epididymis. The analysis of prepubertal (3-week-old) and pubescent (5-week-old) mice show that the CAII protein is detectable during puberty, pointing to specifically regulated expression. This could be a hormonally regulated mechanism that has already been described in rats for CAs (67–69). We propose that CAII enzyme activity plays a major role during puberty to achieve fertility. In contrast to CAII, CAIV is already prepubertally expressed in the corpus epididymidis. However, in the cauda epididymidis, CAIV is initially present in 5-week-old mice. The specific tissue expression might also reflect different functions of CAIV in the male reproductive tract, such as CAIV protein transfer in the corpus epididymidis and sperm storage in the cauda epididymidis.

Generating CAII/CAIV double knockout mice allowed us to demonstrate the importance of both enzymes in sperm for the achievement of fertility. Zhou et al. (70) have described macroscopic anomalies in the rete testis and ductuli efferentes of CAII-deficient mice. This study demonstrates a significantly reduced animal and testis weight in CAII^−/−^ CAIV^−/−^ mice. Detailed macroscopic analyses of the testes indicate that the reduced weight is accompanied by a thinner germinal epithelium. Interestingly, spermatogenesis and sperm storage are not disturbed in CAII^−/−^ CAIV^−/−^ mice. Sperm can be recovered in equal numbers from WT and CAII- and CAIV-null mutant mice despite the significantly reduced height of the germ cell epithelium. It is known that, in the stomach of CAII^−/−^ animals, gene loss is compensated by overexpression of CAIX (71). Analogous results were observed with qRT-PCR analyses in tissue from CAII- as well as CAIV-deficient mice. In comparison with the expression levels in WT tissue, CAIV is overexpressed in all parts of the epididymidis from CAII^−/−^ mice, and the expression of CAII is higher in the cauda epididymidis and vas deferens from CAIV^−/−^ animals. These results were substantiated by relative quantification. RNA expression levels were calculated in relation to the kidney as a control and 18S RNA as an internal control. According to gene and protein expression studies (59), CAXIV, which is localized in the distal part of the epididymidis, might be a putative isoform to compensate for the loss of CAII or CAIV.

Another plausible explanation for the subfertility of CAII^−/−^ CAIV^−/−^ animals is the direct regulatory function of CAII and CAIV in sperm. CAII is a constitutively expressed protein during spermatogenesis and is located in the principal piece of the sperm tail, which explains the high CAII RNA amounts in the testis tissue of WT mice. In contrast, CAIV is a protein that is transferred into the plasma membrane of the entire sperm tail in the corpus epididymidis (65). CAII^−/−^ as well as CAIV^−/−^ (50) mice each display reduced sperm motility and velocity. Using mass spectrometry, we show that CAII and CAIV are the two most important isoforms in murine sperm. The loss of almost all CA activity in sperm of CAII^−/−^ CAIV^−/−^ animals suggests that CAII and CAIV account for most of total CA activity in sperm. As might be expected, CAII^−/−^ CAIV^−/−^ mice exhibit an additional reduction in sperm motility and velocity, and, also, the percentage of immotile sperm was more than doubled compared with WT animals. Two important factors for the regulation of sperm motility are pH and HCO_3_^−^ (31, 72), both of which are regulated by CAs (66). An imbalanced pH and/or HCO_3_^−^ homeostasis seems to be the mechanistic basis for the reduced motility. However, the regulatory mechanisms are complex, species-specific, and not yet well understood (73). Nonetheless, the sperm pH*_i_* of CAII^−/−^ CAIV^−/−^ mice under steady-state conditions is not affected and remains unchanged in physiological buffer. Furthermore, sperm of CAII^−/−^ CAIV^−/−^ animals do not show any defects in intracellular alkalization upon stimulation with ammonium chloride. It is still unclear whether motility dysfunction is caused by an altered fluid composition in the epididymidis or by an unbalanced internal proton regulation in sperm by the loss of CAII and CAIV.

Fortunately, *in vivo* mating experiments provide the most informative findings because CAII^−/−^ CAIV^−/−^ mice are subfertile. The mating of male CAII^−/−^ CAIV^−/−^ mice with WT females results in a drastic decrease in offspring and a reduced fertility of 90% in comparison with pure WT matings. The fertility of CAII^−/−^ CAIV^−/−^ female mice is also affected. One possible cause of the reduced female fertility of 78% could be an altered composition of the uterine fluid (8, 29). In this view, unsuccessful sperm capacitation decreases the fertility potential. The effect of subfertility was even more dramatic when CAII^−/−^ CAIV^−/−^ male and female mice were mated. Only one living litter with one living pub was born. We think that this effect points to the possibility that CAII and CAIV are not only responsible for bicarbonate regulation in sperm but also for effects in the female reproductive tract.

##### CAII and CAIV Are Key Enzymes in the HCO_3_^−^-mediated Beat Frequency Increase during Early Sperm Activation

To prove the involvement of CAII and CAIV in early HCO_3_^−^-mediated events of capacitation, we analyzed sperm motility on a single-cell level. HCO_3_^−^ induces an increase in sperm beat frequency within the first 30 s after application (31). This occurs physiologically when sperm enter the uterus, which is rich in HCO_3_^−^ (29). We are able to increase the sperm beat frequency *in vitro* by stimulating the cells with 15 mm HCO_3_^−^ and 2% CO_2_ (31). In a previous study, we have shown the involvement of CAIV in the HCO_3_^−^-mediated pathway during early sperm activation (50). This study demonstrates that sperm of CAII^−/−^ mice also display a reduced and delayed response to HCO_3_^−^. We conclude that the loss of CAII activity leads to an unbalanced intracellular HCO_3_^−^ homeostasis, which is reflected in the delayed and decreased response to HCO_3_^−^. The findings that CAII and CAIV are the two most important CA isoforms in murine sperm led to the idea of generating CAII^−/−^ CAIV^−/−^ animals to study sperm behavior. To address the question of whether capacitation and late activation of sperm are altered by the loss of CAII and CAIV, we performed a Western blot analysis with sperm of CAII^−/−^ CAIV^−/−^ mice using a phosphotyrosine antibody. As expected, we did not see any changes in tyrosin phosphorylation under capacitating conditions in CAII^−/−^ CAIV^−/−^ compared with sperm of WT animals.

CASA analysis, which tracks the swimming path of the whole sperm population, reveals additional effects on the reduction of motility and velocity in CAII^−/−^ CAIV^−/−^ mice compared with sperm from single CAII knockout mice. Surprisingly, single-sperm beat frequency experiments of motile CAII^−/−^ CAIV^−/−^ sperm do not reveal the expected reduction of response to HCO_3_^−^. In fact, sperm of CAII^−/−^ animals show the greatest delay in response to HCO_3_^−^. One explanation for the counterbalancing of the loss of CAII and CAIV could be that transporters or exchangers adopt the absent CA function, like Na^+^/HCO_3_^−^ co-transporter and Cl^−^/HCO_3_^−^ exchanger (52), or that sperm of CAII^−/−^ CAIV^−/−^ mice compensate for gene loss with the expression of another CA isoform. Such compensatory mechanisms have already been described for CAIX in the stomach of CAII-deficient mice (71). We did not find a compensatory mechanism of CAII and CAIV for each other using Western blot analysis. Post-testicular sperm are translatively inactive, and a possible overexpression of another CA isoform in CAII^−/−^ CAIV^−/−^ sperm is not detectable by qRT-PCR, as is the case in other reproductive tissues. To bypass such a possible genetically induced compensatory mechanism in sperm, the genetic CAII deficiency was combined with a biochemical loss of CAIV by treating sperm of CAII^−/−^ animals with PLC. The response of such CAII^−/−^ CAIV^BC−^ sperm to HCO_3_^−^ or CO_2_ is more delayed and reduced in comparison with the response of the sperm of CAII^−/−^ animals. Because of the fact that HCO_3_^−^ can evolve spontaneously, a response to HCO_3_^−^ without any CA presence is also possible. We conclude that developing sperm possess compensatory mechanisms that help to sustain the essential HCO_3_^−^-mediated pathway during early sperm activation.

In summary, the epididymal localization of CAII and CAIV suggests their involvement in the acidification mechanism of the luminal fluid. In sperm, the catalytic reaction of these two enzymes contributes to nearly 100% of the total CA activity in sperm. They are key enzymes in the regulation of sperm motility and essential for the HCO_3_^−^-mediated beat frequency increase during early sperm activation. Therefore, double knockout of CAII and CAIV leads to subfertility in mice.

## Author Contributions

P. M. W., N. M., J. S., L. G., A. W., H. M. B., W. S. S., and G. W. developed the experimental concepts and performed the experiments. W. S. S. provided CAIV KO animals. P. M. W. and G. W. wrote the manuscript.

## Footnotes

2 The abbreviations used are:

CA: carbonic anhydrase
rb: rabbit
gt: goat
DAB: 3,3′diaminobenzidine
PLC: phospholipase C
CASA: computer-assisted sperm analysis
qRT-PCR: quantitative RT-PCR.

## References

[1] Sullivan R., and Saez F. (2013) Epididymosomes, prostasomes, and liposomes: their roles in mammalian male reproductive physiology. Reproduction 146, R21–35 23613619 10.1530/REP-13-0058

[2] Tulsiani D. R., and Abou-Haila A. (2011) Molecular events that regulate mammalian fertilization. Minerva Ginecologica 63, 103–118 21508901

[3] Lishko P. V., Kirichok Y., Ren D., Navarro B., Chung J. J., and Clapham D. E. (2012) The control of male fertility by spermatozoan ion channels. Annu. Rev. Physiol. 74, 453–475 22017176 10.1146/annurev-physiol-020911-153258 PMC3914660

[4] Visconti P. E., Bailey J. L., Moore G. D., Pan D., Olds-Clarke P., and Kopf G. S. (1995) Capacitation of mouse spermatozoa: 1: correlation between the capacitation state and protein-tyrosine phosphorylation. Development 121, 1129–1137 7743926 10.1242/dev.121.4.1129

[5] Visconti P. E., Moore G. D., Bailey J. L., Leclerc P., Connors S. A., Pan D., Olds-Clarke P., and Kopf G. S. (1995) Capacitation of mouse spermatozoa: 2: protein-tyrosine phosphorylation and capacitation are regulated by a cAMP-dependent pathway. Development 121, 1139–1150 7538069 10.1242/dev.121.4.1139

[6] Dacheux J. L., Belleannée C., Guyonnet B., Labas V., Teixeira-Gomes A. P., Ecroyd H., Druart X., Gatti J. L., and Dacheux F. (2012) The contribution of proteomics to understanding epididymal maturation of mammalian spermatozoa. Syst. Biol. Reprod. Med. 58, 197–210 22788532 10.3109/19396368.2012.663233

[7] Shum W. W., Da Silva N., Brown D., and Breton S. (2009) Regulation of luminal acidification in the male reproductive tract via cell-cell crosstalk. J. Exp. Biol. 212, 1753–1761 19448084 10.1242/jeb.027284 PMC2683015

[8] Miki K., and Clapham D. E. (2013) Rheotaxis guides mammalian sperm. Curr. Biol. 23, 443–452 23453951 10.1016/j.cub.2013.02.007 PMC3607503

[9] Acott T. S., and Carr D. W. (1984) Inhibition of bovine spermatozoa by caudal epididymal fluid: 2: interaction of pH and a quiescence factor. Biol. Reprod. 30, 926–935 6329337 10.1095/biolreprod30.4.926

[10] Kirchhoff C. (1999) Gene expression in the epididymidis. Int. Rev. Cytol. 188, 133–202 10208012 10.1016/s0074-7696(08)61567-3

[11] Okamura N., Tajima Y., Soejima A., Masuda H., and Sugita Y. (1985) Sodium-bicarbonate in seminal plasma stimulates the motility of mammalian spermatozoa through direct activation of adenylate-cyclase. J. Biol. Chem. 260, 9699–9705 2991260

[12] Johnston D. S., Jelinsky S. A., Bang H. J., DiCandeloro P., Wilson E., Kopf G. S., and Turner T. T. (2005) The mouse epididymal transcriptome: transcriptional profiling of segmental gene expression in the epididymidis. Biol. Reprod. 73, 404–413 15878890 10.1095/biolreprod.105.039719

[13] Johnston D. S., Turner T. T., Finger J. N., Owtscharuk T. L., Kopf G. S., and Jelinsky S. A. (2007) Identification of epididymidis-specific transcripts in the mouse and rat by transcriptional profiling. Asian J. Androl. 9, 522–527 17589790 10.1111/j.1745-7262.2007.00317.x

[14] Pholpramool C., Borwornpinyo S., and Dinudom A. (2011) Role of Na+/H+ exchanger 3 in the acidification of the male reproductive tract and male fertility. Clin. Exp. Pharmacol. 38, 403–409 10.1111/j.1440-1681.2011.05525.x 21480944

[15] Kujala M., Hihnala S., Tienari J., Kaunisto K., Hästbacka J., Holmberg C., Kere J., and Höglund P. (2007) Expression of ion transport-associated proteins in human efferent and epididymal ducts. Reproduction 133, 775–784 17504921 10.1530/rep.1.00964

[16] Breton S., Smith P. J., Lui B., and Brown D. (1996) Acidification of the male reproductive tract by a proton pumping (H+)-ATPase. Nat. Med. 2, 470–472 8597961 10.1038/nm0496-470

[17] Kaunisto K. M., and Rajaniemi H. J. (2002) Expression and localization of the Na+/H+ exchanger isoform NHE3 in the rat efferent ducts. J. Androl. 23, 237–241 11868817

[18] Kaunisto K., Parkkila S., Tammela T., Rönnberg L., and Rajaniemi H. (1990) Immunohistochemical localization of carbonic-anhydrase isoenzymes in the human male reproductive-tract. Histochemistry 94, 381–386 2121671 10.1007/BF00266444

[19] Kaunisto K., Parkkila S., Parkkila A. K., Waheed A., Sly W. S., and Rajaniemi H. (1995) Expression of carbonic-anhydrase isoenzymes-IV and isoenzymes-II in rat epididymal duct. Biol. Reprod. 52, 1350–1357 7632842 10.1095/biolreprod52.6.1350

[20] Au C. L., and Wong P. Y. (1980) Luminal acidification by the perfused rat cauda epididymidis. J. Physiol. London 309, 419–427 7252873 10.1113/jphysiol.1980.sp013517 PMC1274593

[21] Parkkila S., Parkkila A. K., Kaunisto K., Waheed A., Sly W. S., and Rajaniemi H. (1993) Location of a membrane-bound carbonic anhydrase isoenzyme (CA IV) in the human male reproductive tract. J. Histochem. Cytochem. 41, 751–757 8468457 10.1177/41.5.8468457

[22] Cordat E., and Casey J. R. (2009) Bicarbonate transport in cell physiology and disease. Biochem. J. 417, 423–439 19099540 10.1042/BJ20081634

[23] Dobyan D. C., and Bulger R. E. (1982) Renal carbonic-anhydrase. Am. J. Physiol. 243, F311-F324 6812435 10.1152/ajprenal.1982.243.4.F311

[24] Jensen L. J., Schmitt B. M., Berger U. V., Nsumu N. N., Boron W. F., Hediger M. A., Brown D., and Breton S. (1999) Localization of sodium bicarbonate cotransporter (NBC) protein and messenger ribonucleic acid in rat epididymidis. Biol. Reprod. 60, 573–579 10026101 10.1095/biolreprod60.3.573

[25] Jensen L. J., Stuart-Tilley A. K., Peters L. L., Lux S. E., Alper S. L., and Breton S. (1999) Immunolocalization of AE2 anion exchanger in rat and mouse epididymidis. Biol. Reprod. 61, 973–980 10491632 10.1095/biolreprod61.4.973

[26] Pastor-Soler N., Piétrement C., and Breton S. (2005) Role of acid/base transporters in the male reproductive tract and potential consequences of their malfunction. Physiology 20, 417–428 16287991 10.1152/physiol.00036.2005

[27] Chang M. C. (1951) Fertilizing capacity of spermatozoa deposited into the fallopian tubes. Nature 168, 697–698 14882325 10.1038/168697b0

[28] Austin C. R. (1951) Observations on the penetration of the sperm into the mammalian egg. Aust. J. Sci. Res. Ser. B 4, 581 14895481 10.1071/bi9510581

[29] Mannowetz N., Wandernoth P., Hornung J., Ruffing U., Raubuch M., and Wennemuth G. (2011) Early activation of sperm by HCO_3_^−^ is regulated hormonally in the murine uterus. Int. J. Androl. 34, 153–164 20500236 10.1111/j.1365-2605.2010.01067.x

[30] Turner R. M. (2006) Moving to the beat: a review of mammalian sperm motility regulation. Reprod. Fert. Develop. 18, 25–38 10.1071/rd05120 16478600

[31] Wennemuth G., Carlson A. E., Harper A. J., and Babcock D. F. (2003) Bicarbonate actions on flagellar and Ca^2+^-channel responses: initial events in sperm activation. Development 130, 1317–1326 12588848 10.1242/dev.00353

[32] Boatman D. E., and Robbins R. S. (1991) Bicarbonate: carbon-dioxide regulation of sperm capacitation, hyperactivated motility, and acrosome reactions. Biol. Reprod. 44, 806–813 1907858 10.1095/biolreprod44.5.806

[33] White D. R., and Aitken R. J. (1989) Relationship between calcium, cyclic AMP, ATP, and intracellular pH and the capacity of hamster spermatozoa to express hyperactivated motility. Gamete Res. 22, 163–177 2540081 10.1002/mrd.1120220205

[34] Curtis M. P., Kirkman-Brown J. C., Connolly T. J., and Gaffney E. A. (2012) Modelling a tethered mammalian sperm cell undergoing hyperactivation. J. Theor. Biol. 309, 1–10 22727894 10.1016/j.jtbi.2012.05.035

[35] Suarez S. S., and Pacey A. A. (2006) Sperm transport in the female reproductive tract. Hum. Reprod. Update 12, 23–37 16272225 10.1093/humupd/dmi047

[36] Demott R. P., and Suarez S. S. (1992) Hyperactivated sperm progress in the mouse oviduct. Biol. Reprod. 46, 779–785 1591334 10.1095/biolreprod46.5.779

[37] Babcock D. F., Wandernoth P. M., and Wennemuth G. (2014) Episodic rolling and transient attachments create diversity in sperm swimming behavior. BMC Biol. 12, 67 25182562 10.1186/s12915-014-0067-3 PMC4354980

[38] Xie F., Garcia M. A., Carlson A. E., Schuh S. M., Babcock D. F., Jaiswal B. S., Gossen J. A., Esposito G., van Duin M., and Conti M. (2006) Soluble adenylyl cyclase (sAC) is indispensable for sperm function and fertilization. Dev. Biol. 296, 353–362 16842770 10.1016/j.ydbio.2006.05.038

[39] Nolan M. A., Babcock D. F., Wennemuth G., Brown W., Burton K. A., and McKnight G. S. (2004) Sperm-specific protein kinase A catalytic subunit Cα2 orchestrates cAMP signaling for male fertility. Proc. Natl. Acad. Sci. U.S.A. 101, 13483–13488 15340140 10.1073/pnas.0405580101 PMC518783

[40] Chen Y., Cann M. J., Litvin T. N., Iourgenko V., Sinclair M. L., Levin L. R., and Buck J. (2000) Soluble adenylyl cyclase as an evolutionarily conserved bicarbonate sensor. Science 289, 625–628 10915626 10.1126/science.289.5479.625

[41] Litvin T. N., Kamenetsky M., Zarifyan A., Buck J., and Levin L. R. (2003) Kinetic properties of “soluble” adenylyl cyclase: synergism between calcium and bicarbonate. Journal Biol. Chem. 278, 15922–15926 12609998 10.1074/jbc.M212475200

[42] Parkkila S., Rajaniemi H., and Kellokumpu S. (1993) Polarized expression of a band 3-related protein in mammalian sperm cells. Biol. Reprod. 49, 326–331 8373956 10.1095/biolreprod49.2.326

[43] Demarco I. A., Espinosa F., Edwards J., Sosnik J., De La Vega-Beltran J. L., Hockensmith J. W., Kopf G. S., Darszon A., and Visconti P. E. (2003) Involvement of a Na+/HCO-3 cotransporter in mouse sperm capacitation. J. Biol. Chem. 278, 7001–7009 12496293 10.1074/jbc.M206284200

[44] Bagnis C., Marshansky V., Breton S., and Brown D. (2001) Remodeling the cellular profile of collecting ducts by chronic carbonic anhydrase inhibition. Am. J. Physiol. Renal Physiol. 280, F437–448 11181405 10.1152/ajprenal.2001.280.3.F437

[45] Carlson A. E., Hille B., and Babcock D. F. (2007) External Ca^2+^ acts upstream of adenylyl cyclase SACY in the bicarbonate signaled activation of sperm motility. Dev. Biol. 312, 183–192 17950270 10.1016/j.ydbio.2007.09.017 PMC2259292

[46] Parkkila S., Kaunisto K., Kellokumpu S., and Rajaniemi H. (1991) A high activity carbonic anhydrase isoenzyme (CA II) is present in mammalian spermatozoa. Histochemistry 95, 477–482 1907954 10.1007/BF00315743

[47] Imtaiyaz Hassan M., Shajee B., Waheed A., Ahmad F., and Sly W. S. (2013) Structure, function and applications of carbonic anhydrase isozymes. Bioorg. Med. Chem. 21, 1570–1582 22607884 10.1016/j.bmc.2012.04.044

[48] Small C. L., Shima J. E., Uzumcu M., Skinner M. K., and Griswold M. D. (2005) Profiling gene expression during the differentiation and development of the murine embryonic gonad. Biol. Reprod. 72, 492–501 15496517 10.1095/biolreprod.104.033696 PMC3217241

[49] Krishnamurthy V. M., Kaufman G. K., Urbach A. R., Gitlin I., Gudiksen K. L., Weibel D. B., and Whitesides G. M. (2008) Carbonic anhydrase as a model for biophysical and physical-organic studies of proteins and protein-ligand binding. Chem. Rev. 108, 946–1051 18335973 10.1021/cr050262p PMC2740730

[50] Wandernoth P. M., Raubuch M., Mannowetz N., Becker H. M., Deitmer J. W., Sly W. S., and Wennemuth G. (2010) Role of carbonic anhydrase IV in the bicarbonate-mediated activation of murine and human sperm. PloS ONE 5, e15061 21124840 10.1371/journal.pone.0015061 PMC2991337

[51] Sly W. S., and Hu P. Y. (1995) Human carbonic anhydrases and carbonic anhydrase deficiencies. Annu. Rev. Biochem. 64, 375–401 7574487 10.1146/annurev.bi.64.070195.002111

[52] Liu Y., Wang D. K., and Chen L. M. (2012) The Physiology of Bicarbonate Transporters in Mammalian Reproduction. Biol. Reprod. 86, 1–13 10.1095/biolreprod.111.096826 22262691

[53] Mannowetz N., Wandernoth P., and Wennemuth G. (2012) Basigin interacts with both MCT1 and MCT2 in murine spermatozoa. J. Cell. Physiol. 227, 2154–2162 21792931 10.1002/jcp.22949

[54] Livak K. J., and Schmittgen T. D. (2001) Analysis of relative gene expression data using real-time quantitative PCR and the 2(-ΔΔC(T)) method. Methods 25, 402–408 11846609 10.1006/meth.2001.1262

[55] Wennemuth G., Babcock D. F., and Hille B. (2003) Calcium clearance mechanisms in mouse sperm. Biophys. J. 122, 115–128 10.1085/jgp.200308839 PMC2234473 12835474

[56] Graber M. L., DiLillo D. C., Friedman B. L., and Pastoriza-Munoz E. (1986) Characteristics of fluoroprobes for measuring intracellular pH. Anal. Biochem. 156, 202–212 3740410 10.1016/0003-2697(86)90174-0

[57] Klier M., Schüler C., Halestrap A. P., Sly W. S., Deitmer J. W., and Becker H. M. (2011) Transport activity of the high-affinity monocarboxylate transporter MCT2 is enhanced by extracellular carbonic anhydrase IV but not by intracellular carbonic anhydrase II. J. Biol. Chem. 286, 27781–27791 21680735 10.1074/jbc.M111.255331 PMC3149368

[58] Ikezawa H. (2002) Glycosylphosphatidylinositol (GPI)-anchored proteins. Biol. Pharm. Bull. 25, 409–417 11995915 10.1248/bpb.25.409

[59] Hermo L., Chong D. L., Moffatt P., Sly W. S., Waheed A., and Smith C. E. (2005) Region- and cell-specific differences in the distribution of carbonic anhydrases II, III, XII, and XIV in the adult rat epididymidis. J. Histochem. Cytochem. 53, 699–713 15928319 10.1369/jhc.4A6575.2005

[60] Saxena D. K., Oh-Oka T., Kadomatsu K., Muramatsu T., and Toshimori K. (2002) Behaviour of a sperm surface transmembrane glycoprotein basigin during epididymal maturation and its role in fertilization in mice. Reproduction 123, 435–444 11882021 10.1530/rep.0.1230435

[61] Schwarz A., Wennemuth G., Post H., Brandenburger T., Aumüller G., and Wilhelm B. (2013) Vesicular transfer of membrane components to bovine epididymal spermatozoa. Cell Tissue Res. 353, 549–561 23715721 10.1007/s00441-013-1633-7

[62] Phelps B. M., Koppel D. E., Primakoff P., and Myles D. G. (1990) Evidence that proteolysis of the surface is an initial step in the mechanism of formation of sperm cell surface domains. J. Cell Biol. 111, 1839–1847 2229175 10.1083/jcb.111.5.1839 PMC2116336

[63] Dacheux J. L., and Dacheux F. (2014) New insights into epididymal function in relation to sperm maturation. Reproduction 147, R27–42 24218627 10.1530/REP-13-0420

[64] Jones R. C., and Murdoch R. N. (1996) Regulation of the motility and metabolism of spermatozoa for storage in the epididymidis of eutherian and marsupial mammals. Reprod. Fertil. Dev. 8, 553–568 8870080 10.1071/rd9960553

[65] Ekstedt E., Holm L., and Ridderstråle Y. (2004) Carbonic anhydrase in mouse testis and epididymidis: transfer of isozyme IV to spermatozoa during passage. J. Mol. Histol. 35, 167–173 15328921 10.1023/b:hijo.0000023387.02793.af

[66] Breton S. (2001) The cellular physiology of carbonic anhydrases. JOP 2, 159–164 11875253

[67] Härkönen P. L., and Väänänen H. K. (1988) Androgen regulation of carbonic anhydrase II, a major soluble protein in rat lateral prostate tissue. Biol. Reprod. 38, 377–384 3129039 10.1095/biolreprod38.2.377

[68] Kaunisto K., Fleming R. E., Kneer J., Sly W. S., and Rajaniemi H. (1999) Regional expression and androgen regulation of carbonic anhydrase IV and II in the adult rat epididymidis. Biol. Reprod. 61, 1521–1526 10569998 10.1095/biolreprod61.6.1521

[69] Caldarelli A., Diel P., and Vollmer G. (2005) Effect of phytoestrogens on gene expression of carbonic anhydrase II in rat uterus and liver. J. Steroid Biochem. Mol. Biol. 97, 251–256 16188437 10.1016/j.jsbmb.2005.05.010

[70] Zhou Q., Clarke L., Nie R., Carnes K., Lai L. W., Lien Y. H., Verkman A., Lubahn D., Fisher J. S., Katzenellenbogen B. S., and Hess R. A. (2001) Estrogen action and male fertility: roles of the sodium/hydrogen exchanger-3 and fluid reabsorption in reproductive tract function. Proc. Natl. Acad. Sci. U.S.A. 98, 14132–14137 11698654 10.1073/pnas.241245898 PMC61180

[71] Pan P., Leppilampi M., Pastorekova S., Pastorek J., Waheed A., Sly W. S., and Parkkila S. (2006) Carbonic anhydrase gene expression in CA II-deficient (Car2^−/−^) and CA IX-deficient (Car9^−/−^) mice. J. Physiol. 571, 319–327 16396925 10.1113/jphysiol.2005.102590 PMC1796798

[72] Babcock D. F., Rufo G. A. Jr., and Lardy H. A. (1983) Potassium-dependent increases in cytosolic pH stimulate metabolism and motility of mammalian sperm. Proc. Natl. Acad. Sci. U.S.A. 80, 1327–1331 6572391 10.1073/pnas.80.5.1327 PMC393590

[73] Nishigaki T., Jose O., Gonzalez-Cota A. L., Romero F., Trevino C. L., and Darszon A. (2014) Intracellular pH in sperm physiology. Biochem. Biophys. Res. Commun. 3, 1149–1158 24887564 10.1016/j.bbrc.2014.05.100 PMC4146485

[74] Badger M. R., and Price G. D. (1989) Isolation and characterization of high CO(2)-requiring-mutants of the cyanobacterium synechococcus PCC7942: two phenotypes that accumulate inorganic carbon but are apparently unable to generate CO(2) within the carboxysome. Plant Physiol. 89, 51–60 16667063 10.1104/pp.91.2.514 PMC1062031

